# An update on the Pharmacological and Psychotherapeutic Management of Treatment Resistant OCD

**DOI:** 10.1192/j.eurpsy.2023.104

**Published:** 2023-07-19

**Authors:** M. Van Ameringen

**Affiliations:** Psychiatry and Behavioural Neurosciences, McMaster University, Hamilton, Canada

## Abstract

**Abstract:**

Although the rates of response to first-line pharmacological treatments (serotonin reuptake inhibitors – SRIs) are generally twice that of placebo, only 40-60% of patients respond sufficiently or are able to tolerate traditional first-line pharmacotherapy. Augmentation with dopamine antagonist medications is associated with the strongest evidence, yet dopamine antagonists benefit only a minority of those who try them and carry elevated risks of adverse effects. Based on evolving pathophysiologic models of OCD, a variety of agents targeting serotonin, dopamine, norepinephrine, glutamate, and anti-inflammatory pathways have been explored as alternative or adjunctive therapies for treatment-resistant OCD and have at least preliminary evidence of efficacy. Similarly, approximately 50% of patients do not respond optimally to first psychotherapeutic treatments including cognitive behavioural therapy (CBT) and exposure and response prevention (ERP), even when combined with pharmacotherapy. The psychotherapy outcome literature is heterogeneous and very few psychological strategies have been developed specifically to treat treatment resistant OCD. However, a recent systematic review concluded that CBT improved treatment response in individuals with pharmacotherapy resistance. This presentation will present an update on the pharmacological and psychotherapeutic treatment of refractory OCD including novel strategies such as the use of psychedelics.

**Disclosure of Interest:**

M. Van Ameringen Grant / Research support from: Elvium, CIHR,Hamilton Academic Health Sciences Organization, Consultant of: Abbvie, Bausch Health, Boehringer Ingelheim, Elvium, Jazz, Vistagen, Speakers bureau of: Abbvie, Elvium (Purdue), Lundbeck, Otsuka, Sunovion, and Takeda

